# *EMC10* homozygous variant identified in a family with global developmental delay, mild intellectual disability, and speech delay

**DOI:** 10.1111/cge.13842

**Published:** 2020-09-15

**Authors:** Muhammad Umair, Mariam Ballow, Abdulaziz Asiri, Yusra Alyafee, Abeer al Tuwaijri, Kheloud M. Alhamoudi, Taghrid Aloraini, Marwa Abdelhakim, Azza Thamer Althagafi, Senay Kafkas, Lamia Alsubaie, Muhammad Talal Alrifai, Robert Hoehndorf, Ahmed Alfares, Majid Alfadhel

**Affiliations:** ^1^ Medical Genomics Research Department King Abdullah International Medical Research Center (KAIMRC), King Saud Bin Abdulaziz University for Health Sciences, Ministry of National Guard Health Affairs (MNGH) Riyadh Saudi Arabia; ^2^ Department of Pathology and Laboratory Medicine King Abdulaziz Medical City Riyadh Saudi Arabia; ^3^ Computer, Electrical and Mathematical Sciences & Engineering Division Computational Bioscience Research Center, King Abdullah University of Science and Technology Thuwal Saudi Arabia; ^4^ College of Computers and Information Technology, Taif University Taif Saudi Arabia; ^5^ Division of Genetics, Department of Pediatrics King Abdulaziz Medical City Riyadh Saudi Arabia; ^6^ Department of Pediatrics, Division of Neurology King Saud Bin Abdulaziz University for Health Sciences, King Abdulaziz Medical City, Ministry of National Guard Health Affairs (MNGH) Riyadh Saudi Arabia; ^7^ Department of Pediatrics College of Medicine, Qassim University Saudi Arabia; ^8^ Division of Genetics, Department of Pediatrics King Abdullah Specialized Children's Hospital, King Abdulaziz Medical City Riyadh Saudi Arabia

**Keywords:** *EMC10*, intellectual disability, speech delay and global developmental delay, splice acceptor site variant

## Abstract

In recent years, several genes have been implicated in the variable disease presentation of global developmental delay (GDD) and intellectual disability (ID). The endoplasmic reticulum membrane protein complex (EMC) family is known to be involved in GDD and ID. Homozygous variants of *EMC1* are associated with GDD, scoliosis, and cerebellar atrophy, indicating the relevance of this pathway for neurogenetic disorders. EMC10 is a bone marrow‐derived angiogenic growth factor that plays an important role in infarct vascularization and promoting tissue repair. However, this gene has not been previously associated with human disease. Herein, we describe a Saudi family with two individuals segregating a recessive neurodevelopmental disorder. Both of the affected individuals showed mild ID, speech delay, and GDD. Whole‐exome sequencing (WES) and Sanger sequencing were performed to identify candidate genes. Further, to elucidate the functional effects of the variant, quantitative real‐time PCR (RT‐qPCR)‐based expression analysis was performed. WES revealed a homozygous splice acceptor site variant (c.679‐1G>A) in *EMC10* (chromosome 19q13.33) that segregated perfectly within the family. RT‐qPCR showed a substantial decrease in the relative *EMC10* gene expression in the patients, indicating the pathogenicity of the identified variant. For the first time in the literature, the *EMC10* gene variant was associated with mild ID, speech delay, and GDD. Thus, this gene plays a key role in developmental milestones, with the potential to cause neurodevelopmental disorders in humans.

## Graphical abstract

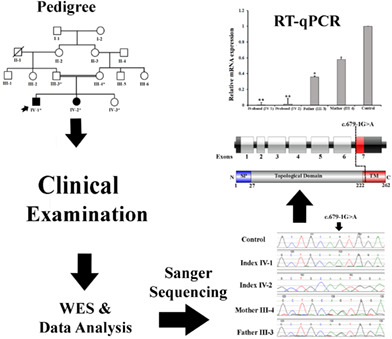

## 1 INTRODUCTION

The endoplasmic reticulum (ER) is a diverse cell organelle whose physical behavior and function greatly impact cell physiology. It plays a key role in different cellular processes, including lipid biosynthesis, membrane and secretory protein biogenesis, Ca^2+^ storage, and metabolic pathway regulation.^1^ ER homeostasis is vital for overall cellular fitness. Therefore, identifying allied pathways and requisite factors is an important goal of modern cell biology.^2^

In yeast, several ER proteins and complexes have been identified in large‐scale genetic interaction studies. However, in most cases, their functions remain unknown.^1^, ^3^ One of these complexes, the ER membrane protein complex (EMC), is an abundant, multi‐subunit protein complex found in all eukaryotic kingdoms.^4^

The EMC family was first identified in yeast as a multi‐protein transmembrane complex composed of 10 subunits, where it was thought to be responsible for eliminating misfolded membrane proteins.^1^ The EMC may facilitate interactions between mitochondria and the ER, thus modulating the processing and folding of different proteins.^5^ It also plays a key role in the stability of different transmembrane proteins, such as rhodopsin, which has been reported to cause retinal degeneration in *Drosophila*.^6^

The loss of ER‐mitochondria crosstalk has been previously associated with Alzheimer's disease (AD) type 3 and 4 (*PSEN1*: OMIM 607822; *PSEN2*: OMIM 600759 [https://www.omim.org/]), recessive cerebellar atrophy, visual impairment, psychomotor retardation (*EMC1*: OMIM 616875), dominant amyotrophic lateral sclerosis (ALS) type 8 (*VAPB*: OMIM 608627), juvenile ALS type 16 (*SIGMAR1*; OMIM 601978), Parkinson's disease type 1 and 4 (*SNCA*: OMIM 163890), and Charcot‐Marie‐Tooth disease (CMT) type 2A2A (*MFN2*; OMIM 608507).^7^ Despite advances in molecular genetic technologies, the function of the EMC and the pathways associated with human disease remain to be fully elucidated.^8^

Herein, we report a single family with two siblings affected by mild ID, speech delay, and GDD. We characterized this clinical phenotype using whole‐exome sequencing (WES) and identified a potential pathogenic homozygous variant in the *EMC10* gene located on chromosome 19q13.33.

## 2 METHODS

### 2.1 Study and ethical approval

**FIGURE 1:**
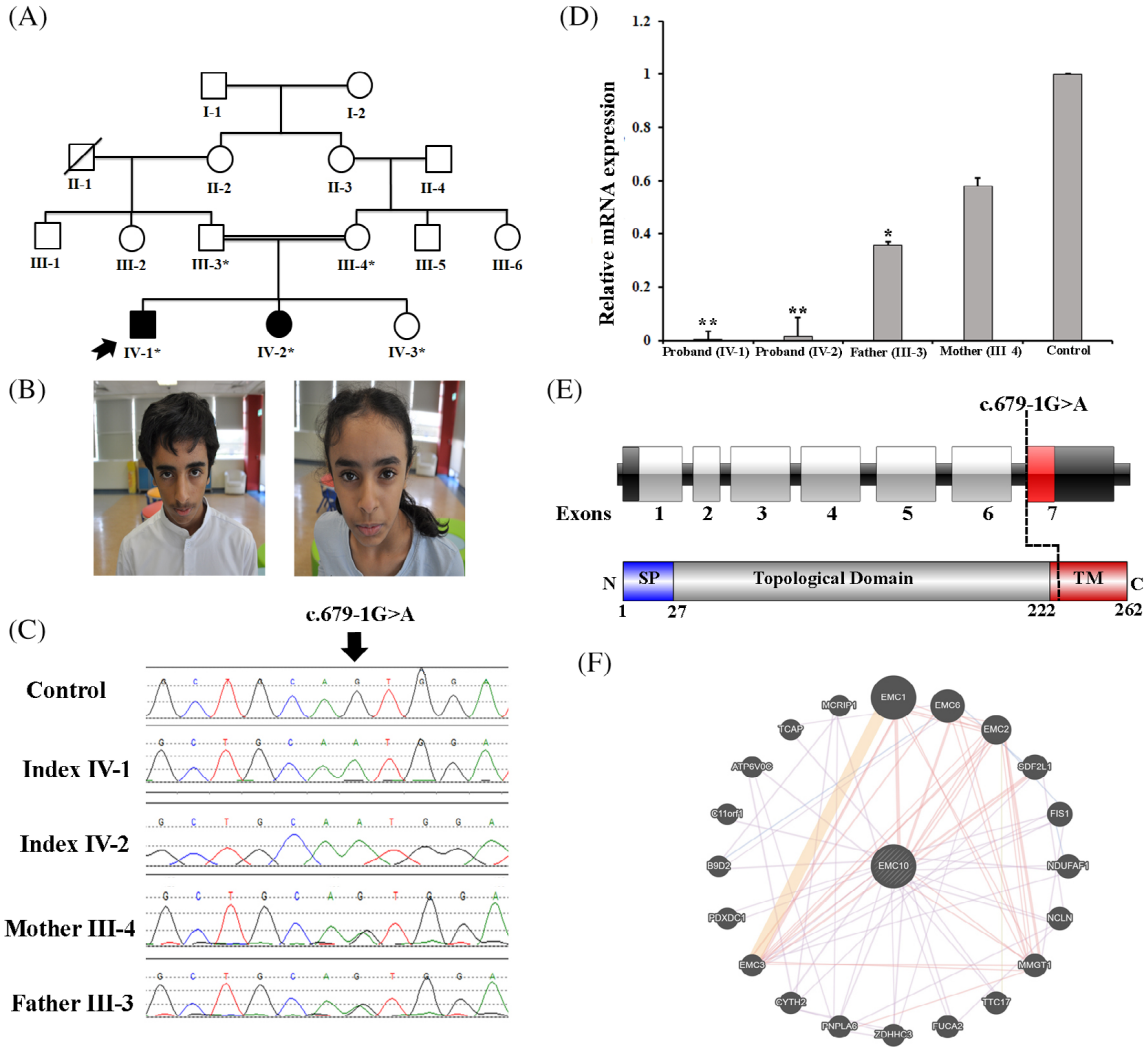
A, Pedigree of the family showing consanguineous union and recessive inheritance pattern. B, Photographs of the affected individual (IV‐1 and IV‐2) exhibiting dysmorphic features, including a triangular face, broad forehead, plagiocephaly, and mild synophrys. C, Sanger sequencing electropherograms of the homozygous affected, heterozygous carriers, and a control sample. D, The *EMC10* gene expression of the affected patients compared to the parents (carriers) and control. The results revealed that the affected individuals had decreased *EMC10* gene expression. E, Schematic representation of the *EMC10* gene with seven exons, the position of variant identified in the present study, and EMC10 protein with N‐terminal signal peptide (1‐27 aa), topological domain (28‐222 aa), and a C‐terminal transmembrane domain (222‐262 aa). F, Schematic representation of the EMC10 interactions with other proteins using GeneMANIA (https://genemania.org/) [Colour figure can be viewed at wileyonlinelibrary.com]

The Institutional Review Board (IRB) of King Abdullah International Medical Research Center (KAIMRC), Riyadh, approved the study with adherence to the Declaration of Helsinki. Written informed consent for the publication of related data was obtained from the parents of the enrolled patient. Fresh blood samples were collected from five individuals, including two affected individuals, a normal sibling, and normal parents (Figure 1A).

### 2.2 Genomic DNA and peripheral blood mononuclear cell extraction

Genomic DNA was extracted from the fresh blood of all available individuals using the QIAamp DNA Micro kit according to the manufacturer's instructions. DNA quantification was performed using a NanoDrop spectrophotometer. Density gradient centrifugation was used to separate peripheral blood mononuclear cells (PBMCs) using Ficoll‐prefilled Leucosep tubes using the standard methods. The percentage of PBMC viability was assessed using the Trypan blue exclusion method (85%‐90%).

### 2.3 Whole exome sequencing

Whole exome sequencing (WES) was performed by Centogene (Rostock, Germany, https://www.centogene.com) for the single affected family member (IV‐1) using an Illumina platform (Illumina, San Diego). Approximately 37 Mb (214 405 exons) of the Consensus Coding Sequence (CCS) were enriched from fragmented genomic DNA using >340 000 probes designed against the human genome (Nextera Rapid Capture Exome, Illumina). The generated libraries were sequenced with an average coverage depth of ×70‐×100.

Data analysis and interpretation were performed by Centogene using an end‐to‐end in‐house bioinformatics pipeline with applications including base calling, filtering of low‐quality reads, and alignment of reads to the GRCh37/hg19 (http://genome.ucsc.edu/) genome assembly.

### 2.4 Filtration of variants

Primary filtering was performed using the standard methods, including filtering out low‐quality reads and potential artifacts. Subsequently, variant annotation was performed using the standard methods. All disease‐causing variants reported in the HGMD, ClinVar, CentoMD, PubMed, and variants with minor allele frequency (MAF) of >1% in the gnomAD/ExAC database were given predilection.

Variant filtration steps also focused on the autosomal recessive mode of inheritance, and coding exons along with flanking ±20 intronic base pairs were given priority. All pertinent inheritance patterns were considered. However, a recessive pattern was given preference based on the pedigree analysis. Patient clinical reports were evaluated to perform genotype‐phenotype correlations. In silico prediction tools were used to analyze the pathogenicity of the identified variants, including NNSplice (Berkeley [https://www.fruitfly.org/seq_tools/splice.html]), FATHMM (http://fathmm.biocompute.org.uk/), DANN (https://cbcl.ics.uci.edu/public_data/DANN/), EIGEN, MutPred Splice (https://genomebiology.biomedcentral.com/articles/10.1186/gb-2014-15-1-r19), SKIPPY (http://research.nhgri.nih.gov/skippy), Human Splice Finder (http://www.umd.be/HSF/), Varsome (https://varsome.com/), and MutationTaster (http://www.mutationtaster.org/).

### 2.5 Sanger sequencing

The identified homozygous variant was Sanger sequenced in all available members of the family. Sanger sequencing was performed as previously described.^9^ Primer pairs were designed using the Primer3 online tool (http://bioinfo.ut.ee/primer3-0.4.0/). Primers included forward (5′‐TCCCTGACCTGTAGTGACGTAG‐3′) and reverse (5′‐CCAAGAAGGGTTTGCTGGTC‐3′) primers (product size, 369 bp; annealing temperature, 60.5°C).

### 2.6 RNA extraction

Total RNA was extracted from PBMCs after the addition of TRIzol reagent (Invitrogen), followed by chloroform (200 μL) for organic and aqueous phase separation. After centrifugation at 4°C for 15 min, RNA in the aqueous phase was transferred into RNase‐free tubes. Washing was performed using isopropanol, followed by precipitation with 75% ethanol. Quantification and purity tests were performed using standard methods.^10^

### 2.7 Quantitative real‐time PCR

Total RNA was extracted to quantitatively monitor the *EMC10* mRNA expression relative to the internal control house‐keeping gene *GAPDH* (glyceraldehyde‐3‐phosphate dehydrogenase; DQ403057). cDNA was synthesized from total RNA using a high capacity cDNA reverse transcription kit (Applied Biosystems). The primer sequences for the *EMC10* cDNA primer pairs can be provided upon request. The primers were designed using the Primerbank database (https://pga.mgh.harvard.edu/Parabiosys/). The qPCR reaction was performed using a SYBR Green Master Mix (Thermo Fisher Scientific, Massachusetts) on a QuantStudio 6 Flex Real‐Time PCR System (Applied Biosystems). No template control (NTC) was used as a negative control for each experiment. All the reactions were repeated independently and performed in triplicate. Data were analyzed using expression Suite software version 1.1 (Applied Biosystems). PCR cycle conditions were the same as described previously,^10^ and GAPDH was used as the endogenous control.

### 2.8 Statistical analysis

The quantitative real‐time PCR results were analyzed using GraphPad Prism (version 8.1). The analysis of variance (one‐way ANOVA) statistical test was applied. *P* < .05 was considered significant.

## 3 RESULTS

### 3.1 Clinical description

#### 3.1.1 Affected individual IV‐1

Patient IV‐1 is a 14‐year‐old male Saudi patient. He was born to consanguineous healthy parents after an unremarkable pregnancy course at full term by cesarean section due to fetal distress. His birth weight was 5 kg (>95th percentile) and the APGAR score was 9 and 9 at 1 and 5 min, respectively. After delivery, the patient was discharged immediately from the hospital.

The patient performed well with normal development until the age of 3, when he started to show speech delay and poor concentration. The patient (IV‐1) developed a febrile seizure at the age of 3.5 during febrile illness, with a body temperature reaching 40°C. The duration of the seizure was short and did not require antiepileptic medication. He started to speak for the first time at the age of 4. Later, at the age of 5, he developed anxiety in the form of separation anxiety and poor sleeping at night. At the age of 10, he started to develop repetitive tics, in the form of repeated blinking, head nodding, eye‐rolling (nystagmus), and jaw tics. His intelligence quotient (IQ) assessment revealed a borderline score of 69.

He was referred to the genetic service at the age of 10 for genetic evaluation. At the age of 10, he started to write his name and was enrolled in a special needs school, with an average academic performance. Currently, the patient has normal gross motor development, with speech delay, since he can speak in sentences but incomprehensively. The patient (IV‐1) has a major deficiency in memory and mathematical abilities.

On examination, he was vitally stable with the following growth parameters: height, 163 cm (50th percentile); weight, 41.2 kg (10th percentile); and head circumference, 56 cm (50th percentile). The patient had subtle dysmorphic features, including a triangular face, broad forehead, plagiocephaly, mild synophrys, and tooth crowding (Figure 1B). Other systemic examinations were unremarkable, except for mild hirsutism.

All the basic metabolic investigations were unremarkable. The karyotype, comparative genomic hybridization (CGH) array, and screening for fragile X were unremarkable. Radiological investigations, including brain MRI and skeletal surveys, were also unremarkable.

#### 3.1.2 Affected individual IV‐2

Patient IV‐2 is an 11‐year‐old female Saudi patient. She is the younger sister of patient IV‐1. She was born after an uneventful pregnancy course at full term by cesarean section due to fetal distress and a large fetus size. Her birth weight was 3.5 kg (50th percentile), with a length of 52 cm (75th percentile) and a head circumference of 36 cm (90th percentile). The APGAR scores were 6 and 8 at 1 and 5 min, respectively. After delivery, she was discharged immediately from the hospital.

At the age of 2, the family noticed symptoms comparable to those of her affected brother (IV‐1), such as speech delay. Later, she developed anxiety in addition to some visual illusions, mainly at night, as well as poor sleep. She had stereotypic movements in her hands and repeated tics, including blinking and nystagmus (rapid eye movements, side to side). She had a friendly personality, no aggressive behavior, and no history of seizures.

She started to walk around 18 months. She started to write her name after the age of 10. However, she still has speech delay, incomprehensive speech, and dysarthria. She is attending a special needs school and has major deficiencies in memory and mathematical abilities. Her IQ test score was 76, which was better than that of her brother (IV‐1).

On examination, she was vitally stable and her latest growth parameters were within normal limits: height, 144 cm (50th‐75th percentile); weight, 34.1 kg (25th‐50th percentile); head circumference, 53 cm (50th percentile). She had subtle dysmorphic features, such as triangular face, frontal bossing, mild synophrys, and tooth crowding (Figure 1B). Other systemic examinations were unremarkable, except for mild hirsutism. Clinical descriptions of both affected individuals are provided in Table 1.

**TABLE 1:** Clinical description of patients IV‐1 and IV‐2

Clinical phenotypes	IV‐1	IV‐2
Sex	Male	Female
Origin	Saudi	Saudi
Consanguinity	+	+
Pregnancy event	Uneventful full term	Uneventful full term
Age at last exam	14	11
Genetic results	c.679‐1G>A in *EMC10* gene	c.679‐1G>A in *EMC10* gene
Global developmental delay	+	+
Speech delay	+	+
Mild‐Intellectual disability	+	+
IQ score	69	76
Anxiety	+	+
Poor sleep	+	+
Repetitive tics	+	+
Major deficiency in memory and mathematical abilities	+	+
Head circumference	56 cm (50th percentile)	53 cm (50th percentile)
Height	163 cm (50th percentile)	144 cm (50th‐75th percentile)
Weight	41.2 kg (10th percentile)	34.1 kg (25th‐50th percentile)
Dysmorphic features	+	+
MRI brain	Normal	Normal
Skeletal survey	Normal	Normal
Hearing test	Normal	Normal
Eye exam	Normal	Normal
Echocardiogram	Normal	Normal

### 3.2 Whole‐exome sequencing and Sanger sequencing

WES was performed for single affected individuals (IV‐1), as described previously.^11^, ^12^ Filtration of the identified variants was performed considering all patterns of inheritance. We focused only on pathogenic, likely pathogenic, variant of uncertain significance (VUS), and non‐synonymous (NS) variants causing missense, nonsense, frame‐shift, splice site variants (SS), coding insertions, or deletions (indel).

The outcomes of WES data analysis and the filtration steps resulted in the identification a homozygous splice acceptor site variant (c.679‐1G>A; g.50985405G>A) in intron 6 of the *EMC10* gene (NM_206538.4; NC_000019.10), located on chromosome 19q13.33 (GRCh37), which segregated perfectly with the disease phenotype, as verified by bi‐directional Sanger sequencing (Figure 1C). The pathogenicity index of the identified variant (c.679‐1G>A) was calculated using different available online tools and was considered disease causing. This *EMC10* variant was validated using the standard methods and screened in different online databases. It was not observed in the homozygous state in the ExAC (http://exac.broadinstitute.org/), gnomAD (http://gnomad.broadinstitute.org/), dbSNP (https://www.ncbi.nlm.nih.gov/projects/SNP/), 1000 Genomes Project (http://www.internationalgenome.org/), or in‐house 2000 exome database. It was classified as a Class 3 VUS, according to the recommendations of Centogene and ACMG.

### 3.3 Quantitative PCR

Using RT‐qPCR, *EMC10* mRNA expression in the PBMCs was inspected in both the affected individuals, parents, and control individuals. The qPCR data revealed that both affected individuals (IV‐1, IV‐2) with the homozygous variant (c.679‐1G>A) showed a substantial reduction in the relative gene expression of the human *EMC10* gene compared to the carrier parents (III‐3, III‐4) and control (IV‐3) (Figure 1D).

## 4 DISCUSSION

The ER membrane protein complex (EMC) was first discovered in yeast as a 6‐subunit (Emc1‐6) transmembrane protein complex, which helps in proper ER protein folding. Subsequently, 10 subunits (EMC1‐10) were observed in different animal species.^1^, ^13^ EMC is an evolutionarily conserved and multifunctional protein complex with a significant functional importance in ER‐mitochondria tethering, ER‐associated degradation, and the proper assembly of different transmembrane proteins.^12^ EMC6 is reported to regulate cell autophagy in humans, and modulates the ER‐associated degradation of particular proteins in *Caenorhabditis elegans*.^14^, ^15^

Pathogenic homozygous variants in the *EMC1* gene have been associated with a recessive neurodevelopmental disorder, characterized by pathognomonic GDD, cerebellar atrophy, ID, visual impairment, psychomotor retardation with epilepsy, and scoliosis.^16^, ^17^ EMC1 also belongs to the EMC family of proteins and interacts with EMC10 (Figure 1F). Here, we investigated two Saudi patients with hallmark features of anxiety disorder, speech delay, tics, and mild ID. Using WES following Sanger sequencing, we identified a homozygous splice acceptor site variant (c.679‐1G>A) in the *EMC10* gene located on chromosome 19q13.33, consisting of a total of seven exons, encoding a 262‐amino acid protein in humans (Figure 1E). The identified variant is located in intron 6 of the *EMC10* gene. As the splice site variant involving ±1 or 2 bases of an intron has a strong impact on the normal splicing process of the gene, the variant identified here (c.679‐1G>A) may affect the canonical splicing of exon 7 by knocking down its natural 3‐acceptor splice site and eliciting the use of a cryptic splice site downstream of the intron. Furthermore, the RT‐qPCR data revealed a substantial decrease in the mRNA expression of the *EMC10* mutant compared to the other family members and control sample.

In the Decipher database (https://decipher.sanger.ac.uk/, Deciphering Developmental Disorders project), two additional cases of variants located in the loci of ECM10 have been reported. The first is a homozygous biparental frameshift variant located in exon 4 of the *EMC10* gene. The phenotypes associated with these variants include abnormalities in different systems, such as nervous system, skeletal system, head or neck, digestive system, and limbs (https://decipher.sanger.ac.uk/search/ddd-research-variants/results?q=emc10).

Since our knowledge of EMC biology is limited, the proper functional role of EMC and its association with human diseases remain to be fully elucidated. However, it has been suggested that EMC10 is a secreted protein and is found in pancreatic beta cells. The *EMC10* gene is regulated by glucose, indicating its key role in glucose metabolism.^18^ In an in vitro study, EMC10 was suggested as a potential therapeutic target for malignant glioblastoma after being found to exert cell proliferation inhibition, invasion, angiogenesis in endothelial cells, and cell migration in glioma cell lines.^19^, ^20^ In schizophrenia mouse models, reduced Mirta22 (human EMC10 ortholog) levels completely rescued the dendritic deficits and spine formation at the hippocampal pyramidal neurons, thus suggesting a key role in neuronal dendrites and spine development.^21^ Furthermore, studies revealed that knockout mouse (*emc10* ^*−/−*^) showed effects on their behavior (hyperactivity, abnormal gait, and abnormal vocalization), decreased bone mineral content, persistence of hyaloid vascular system, cardiovascular issues (decreased heart rate), deceased corpuscular volume in females, thrombocytopenia, metabolic effects, and infertility phenotypes in males (MGI: 5548589).^22^

The EMC has been associated with protein folding and ER‐mitochondria crosstalk, and variants in *EMC* genes such as *EMC1* have been previously associated with syndromic neurodegeneration.^5^, ^8^, ^23^, ^24^, ^25^ Variants in the *COPA* genes (OMIM 601924) have been associated with impaired intracellular communication, which resulted in impaired inter‐organelle transport, increased ER stress, and cytokine generation, causing hereditary autoimmune lung disease and arthritis.^26^ Similarly, variants in *TANGO2* (OMIM 616878) have been associated with recurrent metabolic encephalomyopathic crises with rhabdomyolysis, cardiac arrhythmias, and neurodegeneration, resulting due to vesicular Golgi‐ER transport and an increased ER stress, highlighting the importance of maintaining proper intracellular organelle crosstalk.^27^, ^28^

In conclusion, this study is the first to define the phenotypic spectrum of *EMC10*‐associated disorders, including GDD, mild ID, and speech delay. Our work provides evidence that homozygous variants in *EMC10* may lead to neurodevelopmental disorders in humans. However, further study will be needed to identify the exact mechanism, which may involve the dysregulation of ER–mitochondria tethers or proper protein folding.

## CONFLICT OF INTEREST

The authors declare no conflict of interest.

## AUTHOR CONTRIBUTIONS

Umair M. drafted the manuscript. Ballow M. performed the experiments. Asiri A., Alhamoudi K., Aloraini T., Abdelhakim M., Althagafi T.A., Kafkas S., Alsubaie L., and Alyafee Y. analyzed the data. al Tuwaijri A. performed analysis and Sanger sequencing. Alrifai M.T. edited the manuscript and participated in patient clinical management. Hoehndorf R., Alfares A., and Alfadhel M. edited the manuscript, analyzed the data, participated in patient clinical management, and supervised the work. Umair M. and Alfadhel M. conceptualized and designed of the research.

## ETHICS STATEMENT

The study was approved by the research committee of the King Abdullah International Medical Research Center (KAIMRC) in Riyadh, Saudi Arabia.

## Data Availability

Data will be provided by the corresponding author upon potential requests.
